# Effect of dietary interventions during weaning period on parental practice and lipoproteins and vitamin D status in two-year-old children

**DOI:** 10.1080/16546628.2017.1350127

**Published:** 2017-07-14

**Authors:** Nina Cecilie Øverby, Sigrunn Hernes, Margaretha Haugen

**Affiliations:** ^a^ Department of Public health, Sport and Nutrition, University of Agder, Kristiansand, Norway; ^b^ Department of Environmental Exposure and Epidemiology, Norwegian Institute of Public Health, Oslo, Norway

**Keywords:** Cooking classes, lipoproteins, toddlers, feeding practices, diet

## Abstract

**Objective**: Evaluate if a two-day course for parents on nutrition and applied baby food preparation had an effect on child’s intake of home-made foods, lipid concentration, and vitamin D status.

**Design**: Randomized controlled trial at age 6 months and follow-up at ages 15 and 24 months.

**Setting**: Four health care clinics in Kristiansand, Norway.

**Subjects**: Thirty-nine pairs of 6-month-old children and their parents in the intervention group and 20 pairs in the control group.

**Results**: At age 15 months, the intervention group had lower intakes of ready-made porridge (2.0 vs. 5.8 servings per week (*p* < 0.05)), lower intake of canned baby food (2.9 vs. 6.3 servings per week (*p* < 0.05)) and higher intakes of home-made porridge (4.8 servings vs. 0.9 servings per week (*p* < 0.001)) compared with the control group. The intervention group had higher HDL cholesterol concentrations at 2 years than the control group, 1.08 mol/l compared to 0.89 mol/l (*p* < 0.05).

**Conclusions**: This is the first study to show that providing dietary information and applied baby food preparation to parents during the weaning period may have impact on the children’s diet at 15 and 24 months and improve their lipid profile. Our results call for studies with more power and longer follow-up.

## Introduction

There has been a decrease in the time spent on cooking and home preparation of family meals over the last few decades [1–3]. This shift has been in parallel with increases in intake of energy- and calorie-dense foods with low nutritional value [4,5]. Some previous studies have suggested that children whose parents cook their meals from scratch have a higher nutrient quality, and have higher intakes of fruits and vegetables [6,7]. A healthier diet in children aged 6–13 years seems to correlate with a higher frequency of family meals and home-prepared food [8]. Parents are the providers of the child’s diet, and therefore their ability to prepare healthy meals is critical to their child’s health [9].

Growth in infancy and childhood can have lasting effects on adult health [10]. Childhood obesity increases the risk of multiple acute and chronic medical and psychological problems that can persist into adulthood and adversely affect quality of life and overall life expectancy [11]. Norway has lower rates of paediatric obesity than other developed countries, but data from the Norwegian Child Growth Study (2008–2012) showed that in 8-year-old children 1 in 6 is overweight or obese and 8% have abdominal obesity [12]. In the same study, researchers showed that rapid BMI increase during early life may be associated with a higher risk of obesity at 8 years, highlighting the importance of early growth [13]. The development of prevention strategies to control obesity has proven difficult, as obesity has a multifactorial aetiology, with genetic, physiological, metabolic, environmental and behavioural factors [14]. Addressing life-long dietary intake and especially diet in the first years of life is a suggested strategy [15].

Diet is one of the dominant causes of the burden of disease including non-communicable diseases in Norway [16]. The effect of dietary intake during infancy on cardiovascular disease (CVD) risk is not clear, but it has been suggested that breastfeeding may program for lower total serum cholesterol (TC) and LDL cholesterol in adulthood [17]. It has also been shown that dietary fat quality has a considerable effect on serum lipid concentration in early childhood and into adulthood [18,19].

In Norway, there are dietary guidelines for infants that focus on exclusive breastfeeding for 6 months, introducing vitamin D supplements from 4 weeks of age, and introducing a variety of foods from age 6 months [20]. Parental beliefs and understanding are crucial determinants of infant feeding and behaviour [21]. New parents deal with numerous challenges and parenthood might be overwhelming for some. All mothers and fathers want to serve their children nutritious food, but uncertainty about infant feeding and weaning food makes it easy for parents to choose convenience baby food like porridges, dinners and fruit purees that are readily available in grocery stores [21,22]. In Norwegian infants, more than 80% stated in 2004 that they used ready-made porridge for their babies at the age of 1 year [23]. Improving nutritional awareness and reassure parents of basic food preparation can prove to be of value for nutritional status of the child [24,25] and thereby optimize growth and reduce the risk of overweight.

The main objective of this study was to evaluate whether a two-day parental course with home-made baby food preparations and introduction to nutrition during the weaning period would have an effect on child food intake, lipid concentration and vitamin D status at 15 and 24 months.

## Methods

### Study design

This randomized controlled trial was performed in 2012–2015. The parents of 4–6-month old infants were randomly allocated into intervention and control groups. The intervention consisted of a two-day cooking class. Follow-up evaluations of both the intervention group and the control group were performed when the children were 15 and 24 months old, where blood was drawn from the child’s index finger. Parents completed a questionnaire when the child was 6, 15 and 24 months old. At the end of the study, a mother from the intervention group and one from the control group each won a prize of 5,000 NOK for study completion.

### Subjects

**Figure 1.**
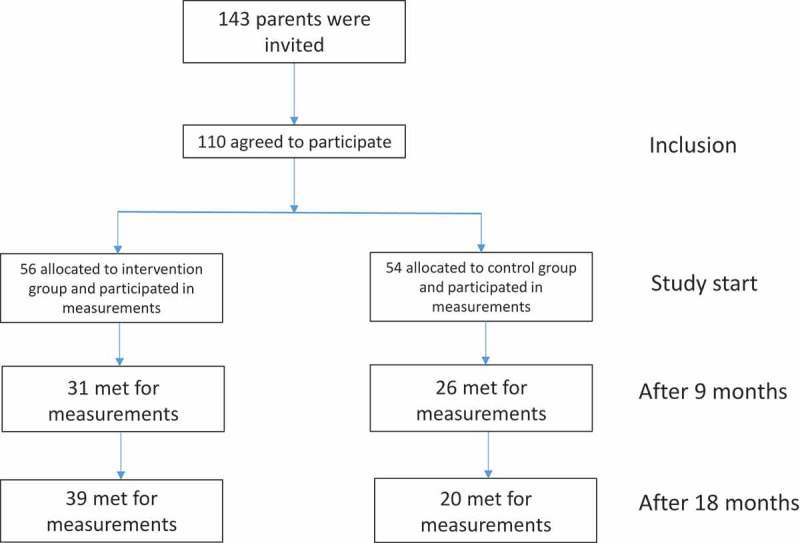
Flowchart of parental participation.

One hundred and forty-three parents attending the 6-month baby check-up at four health care clinics, representing different socioeconomic areas, in Kristiansand, Norway were invited to participate in the study Figure 1. The participation rate was 77%, with 110 parents signing informed consent.

### Description of the intervention

The intervention consisted of two course days which lasted for 4 hours, and parents were given nutritional information and instruction on how to prepare nutritious and varied dishes. The theoretical themes were selected to amplify the Norwegian dietary recommendations for infants [20] and included the following themes: importance of a varied diet, regular meals and development of food preferences and ways to reduce risk of food neophobia. Importance of iron content in the infant diet and vitamin D supplements were also mentioned. The guidelines include a separate information pamphlet on how one may make the baby’s food from scratch. Both courses started with the theory described and were delivered by a home-economics teacher and a Master’s student in public health. The two course days had different focus. The first day focused on food typically eaten for breakfast and lunch in Norway. The participants prepared various kinds of fruit purées, porridges, breads, and nutritious spread. The fruit purees varied according to season and time of the year, containing fresh fruit and berries such as raspberries, blackcurrants, pears, apples, nectarines and dried apricots (without preservatives) and prunes. Porridge was made of millet, oats, spelt and whole wheat flour. The second day focused on nutritious dinners. The participants prepared purées of carrots, potatoes, broccoli, sweet potatoes, cauliflower, avocado and rutabaga. Purées of vegetables with tomatoes and cheese, chicken and tuna dishes with puréed peas were also introduced. The recipes were selected based on recipes that the Danish Health Authorities recommends for infants and toddlers [26], and all food items were easily accessible in Norway. The participants were given written pamphlets with the recipes. The intervention courses started in November 2012 and were given to five groups at two different times. The cooking classes were restricted to 8–14 participants in each group and were carried out at the kitchen laboratories of the University of Agder, Kristiansand, Norway.

Women allocated to the control group were given standard dietary information from midwifes.

#### Assessment methods

##### Food consumption

Child food consumption was assessed with food frequency questionnaires at ages 6, 15 and 24 months with age-specific questions. Questions on breastfeeding were posed in the 6- and 15-month questionnaire: ‘How long has your child been exclusively breastfed (number of months)?’, ‘Is your child breastfed now?’ The response options were: ‘Yes’, ‘No, but the child was breastfed previously’, and ‘No, the child has never been breastfed’.

The use of cod liver oil and vitamin D drops was asked in all three questionnaires: ‘How often (times per week or times per day) does your child take cod liver oil and vitamin D drops?’ The response options were in a range including: ‘never’, ‘less than once per week’ and ‘twice a day or more’.

Frequent use of convenience and self-prepared baby foods was questioned. The response options were in a range from ‘never/seldom’ to ‘four times per day or more’.

##### Anthropometric measurements

The child’s weight and height at birth and at 6, 15 and 24 months were recorded in the health care clinics and transferred to our dataset. Body mass index (BMI) was calculated by weight (kg)/(height (m))^2^ and categorized into normal weight, overweight and obese and according to the International Obesity Task Force cut-offs at 2 years [27]. Weight gain velocity (weight gain/month) between 6 and 24 month of age was calculated by dividing weight gain by the number of months between the two appointments.

##### Blood samples

At the ages of 15 and 24 months, finger blood tests were taken from the children in both the intervention and control group. A competent and comforting nurse drew the blood from the child’s fingertip. Samples were centrifuged in an Eppendorf Centrifuge 5416 within 10 minutes. The tests were stored for up to 7 days at 0–4°C in Kristiansand and transported to Oslo for analysis at the certified chemical analysis lab VITAS.

Serum samples were analysed for HDL, LDL/VLDL cholesterol and 3-epi-25OH vitamin D3. In total, 69 and 59 blood samples were taken at ages 15 months and 24 months, respectively. HDL and LDL/VLDL were separated by PEG precipitation and then cholesterol concentrations were determined using a single working reagent that combines cholesterol ester hydrolysis, oxidation and colour reaction in one step. The colour intensity of the reaction product at 570 nm is directly proportional to HDL or LDL/VLDL cholesterol concentration in the sample. All endpoint measurements were done using either a plate reader with a 570-nm filter or a monochromator.

Determination of 25OH vitamin D3 and 3-epi-25OH vitamin D3 in plasma was done with HPLC-APCI-MS/MS. Forty microlitres of human plasma were diluted with 120 µl isopropanol with deuterium-labelled 25OH-vitamin D3 as the internal standard. After thorough mixing (10 min) and centrifugation (20 min, 4000 × *g* at 10°C), an aliquot of 30 µl was injected from the supernatant into the HPLC system. HPLC was performed with an Agilent 1260/1290 liquid chromatograph (Agilent Technologies, Palo Alto, CA, USA) and interfaced by atmospheric pressure chemical ionization (APCI) to an Agilent Technologies 6420 Triple Quad LC-MS/MS operated in multiple reaction monitoring mode (MRM). Vitamin D analogues were separated on an Ascentis® Express F5 150-mm × 4.6-mm column with 2.7-µM particles. The column temperature was 20°C. A one-point calibration curve was made from analysis using a natural plasma calibrator, where the values were set using the reference materials from Chromsystems (lot no: 1111). Recovery is 95%, the method is linear from 5 to 250 nM at least and the limit of detection is 3 nM for 25OH-vitamin D2 and 5 nM 25OH-vitamin D3. RSD is 7.7% (135.9 nM) and 7.9% (65.1 nM).

#### Statistical analyses

Mean and (SD) were used to describe demographics, serum vitamin D and lipid concentrations. Independent sample *t*-test was used to test between group differences. Linear regression was used to evaluate the effect on HDL concentration at 24 months of age. To evaluate the effect of food intake on HDL concentration, the frequency of self-made porridge intake was added into the linear regression model. In addition, we adjusted for age, sex and BMI at 2 years, but these variables were not significant in the models and changed the estimate by less than 10%, and are therefore not included in the analyses. *P*-values < 5% were considered significant. Statistical analyses were performed using SPSS 23.0 (SPSS, Chicago, IL, USA).

## Results

Baseline characteristics of the study population completing the study are seen in Table 1. Six children in the intervention group and two children in the control group were born preterm. The birthweight was significantly lower in the preterm children compared with the full-term children. However, at inclusion in the study there was no difference in body weight between the groups (Kruskal–Wallis *h*-test), indicating early catch-up growth in the preterm children.

**Table 1.** Demographics at inclusion of 39 mother–child pairs in the intervention group and 20 mother–child pairs in the control group.

	Intervention group *n* = 39	Control group *n* = 20
Age at inclusion (months), mean (SD)	6.2 (0.9)	6.4 (2.2)
Weight at inclusion (kg), mean (SD)	7.7 (0.9)	7.9 (0.9)
Length at inclusion (cm), mean (SD)	67.8 (2.8)	68.5 (2.8)
Birth weight (g), mean (SD)	3388 (561)	3516 (404)
Length at birth (cm), mean (SD)	50.0 (2.6)	50.0 (4.4)
Borne preterm n (%) – before week 37	4 (10.3)	2 (10.0)
Borne preterm n (%) – before week 36	2 (5.1)	0 (0.0)
Number of children with siblings, *n* (%)	8(21)	8(40)
Any breastfeeding, *n* (%)	24 (62.0)	12 (60)
Introduced food at *n* (%)	38 (97)	19 (95)
Age of mother, years (SD)	30.0 (4.0)	31.3 (3.7)
Age of father, years (SD)	32.1 (5.2)	32.2 (3.3)
Education mother, *n* (%)		
≤12 years	0	0
13–16 years	4 (10)	2 (10)
≥17 years	34 (87)	18 (90)
Missing	1 (3)	0
Education father, *n* (%)		
≤ 12 years	1 (3)	1 (5)
13–16 years	10 (26)	5 (25)
≥17 years	26 (66)	14 (70)
Missing	2 (5)	0
Civil status mother, *n* (%)		
Married/cohabitant	38 (98)	20 (100)
Mother smoked at inclusion – no	38 (97)	20 (100)
Father smoked at inclusion – no	36 (92)	18 (90)

Of the 110 parent/child pairs recruited to the study, 56 were randomly allocated to the intervention group, and 54 to the control group. The appointment at age 15 months was missed by 26 (50%) in the control group and 10 (18%) in the intervention group, and 31 (57%) and 15 (27%) missed the appointment at age 24 months in the control and intervention groups, respectively. Reasons for non-attendance for the intervention group were: moving to other cities, long travel to examination site, toddlers being sick, and parents worrying about the blood test. In the control group many had in addition forgotten that they were still participating in the study or felt no responsibility to follow up because they had not participated in the cooking classes. The final numbers of children who completed the study were 39 in the intervention group and 20 in the control group (Figure 1).

Frequent use of convenient porridge was significantly lower in the intervention group compared with the control group at age 15 months (*p* = 0.004), but not at age 24 months. Use of self-made porridge was significantly more frequent in the intervention group compared with the control group at 15 months of age (*p* < 0.001). Frequent use of low-fat milk was higher in the intervention group compared with the control group, although not significantly (*p* = 0.14), while use of convenience dinners were lower at 15 and 24 months of age (*p* = 0.01 and 0.07, respectively) in the intervention group compared with control group. Frequent intake of meat and fish dinners was similar in the two groups. There was no significant difference at 24 months between the two groups regarding weight and height (Table 2). Two children were classified as overweight, one in the intervention group and one in the control group.

**Table 2.** Anthropometric measurements at inclusion and at 15 and 24 months among the 39 children in the intervention group and 20 in the control group who completed the study. Blood lipids concentrations are shown for blood drawn at 15 and 24 months.

	Intervention group inclusion *n* = 39	Control group inclusion *n* = 20	Intervention group 15 months *n* = 38	Control group 15 months *n* = 19	Intervention group 24 months *n* = 39	Control group 24 months *n* = 20
Age (months), mean (SD)	6.2 (.9)	6.4 (2.2)	16.1 (2.1)	15.9 (1.4)	26.1 (2.1)	25.3 (2.2)
Gender, boys, *n* (%)	18 (46.2)	12 (60.0)	18 (47.4)	12 (63.2)	-	-
Weight (kg), mean (SD)	7.6 (0.9)	7.9 (1.0)	10.2 (1.2)	10,8 (1.3)	12.6 (1.4)	12.8 (1.1)
Length (cm), mean (SD)	67.8 (2.8)	68.5 (2.8)	78.2 (3.1)	80.8 (4.1)	88.2 (3.8)	88.3 (3.5)
Breastfed yes, *n* (%)	24 (62.1)	12 (60.0)	7 (17.9)	7 (36.8)	2 (5.1)	1 (5.)
BMI (kg/m^2^)					16.1 (1.3)	16.5 (1.2)
Weight gain from 6 to 24 months of age (g/month)					246.3 (52.7)	267.4 (60.6)
Use of cod liver oil, *n* (%)			18 (46.2)*	3 (15.0)	19 (48.7)*	3 (15.0)
Use of other vitamin D supplement, *n* (%)			8 (20.5)	4 (20.0)	6 (15.4)	0
Mean servings of home-made porridge per week and per child			4.8(5.2) **	0.9 (1.9)	3.0 (3.9)	1.6 (2.4)
Mean servings of convenience porridge per week and child			2.0 (4.0)*	5.8(8.3)	0.4 (1.1)	0.7 (2.9)
Mean servings of canned food per week and child			2.9 (4.1)*	6.3(7.6)	0.6(1.8)	1.8(3.2)
25OHD3 (mol/l) mean (SD)			75.6 (22.7)	77.0 (25.7)	66.2 (17.2)	62.7 (21.8)
HDL-chol. (mol/l) mean (SD)			0.81 (0.33)	0.94 (0.32)	1.08 (0.30)*	0.89 (0.24)
LDL and VLDL-chol. (mol/l), mean (SD)			1.6 (0.5)	1.8 (0.5)	2.0 (0.6)	2.2 (0.4)

* *p* < 0.05 and **p < 0.001 Mann–Whitney *U*-test.

Use of vitamin D supplementation was significantly higher in the intervention group, and the concentration of 25OH-vitamin D was 66.2 mol/l in the intervention group and 62.7 mol/l in the control group (not significantly different between the two groups). There was an overall reduction in the concentration of vitamin D from 15 to 24 months of age, although not significantly (Table 3).

**Table 3.** Differences in blood concentrations from 15 to 24 months of age.

Substance	Intervention group *N* = 32	Control group *N* = 19	*p*-value^a^
25OHD3 mmol/l mean (SD)	−7.0 (13.9)	−15.7 (15.4)	0.057
HDL-chol. mmol/l mean (SD)	0.29 (0.41)	−0.08 (0.40)	0.002
LDL/VLDL mmol/l mean (SD)	0.40 (0.834)	0.34 (0.67)	0.785

^a^Mann–Whitney *U*-test.

While the control group had a non-significant reduction in the HDL cholesterol concentration (−0.08 mmol/l), the intervention group had a significant increase (0.29 mmol/l) (*p* = 0.001, Student *t*-test) from 15 to 24 months of age (Table 3). The concentration was significantly higher in the intervention group (1.08 mmol/l) compared with the control group (0.89 mmol/l) at 24 months (*p* = 0.023) (Table 2). Frequent use of self-made porridge was positively associated with HDL concentration at 24 months of age (β = 0.02 (0.00, 0.04), *p* = 0.03).

## Discussion

This study shows that a cooking intervention for parents during infants’ introduction to solids affects the child’s later diet and lipid profile.

Previous research suggests that cooking food from scratch can improve diet quality [9,28]. The societal decline in cooking skills in recent decades makes it difficult for parents to prepare healthy foods for their children, and many rely on industrially produced baby food. To our knowledge, no previous cooking intervention study in parents of infants has reported the effects on child diet. However, Garcia et al. reported that parental diet was improved after cooking course participation [29]. The children in the intervention group in the present study had a lower intake of canned baby food at 2 years of age and lower intakes of ready-made porridge at 15 months of age. They also had higher intakes of home-made porridge at age 15 months. The intervention focused on how parents could make porridge and dinners themselves for their children. These results show that the intervention was effective in this respect. As more than 80% of Norwegian infants mainly eat industrially produced food, there is a concern about food variety in young children. Garcia and colleagues reported that the UK infant food market supplies mainly sweet, soft, spoonable foods targeting infants from age 4 months, and that these foods do not serve the intended purpose of increasing nutrient density in children’s diets [30]. Another study by Garcia and colleagues [31] reported that the fruit and vegetables used in industrially produced baby foods consists of fruits and relatively sweet vegetables that do not encourage preferences for bitter tastes, which is important for increasing the intake of vegetables. Our study shows a decrease in canned food and may in that relation improve food variety.

Vitamin D is especially important during periods of growth, as in infancy, due to bone mineral accrual [32]. Vitamin D supplements are recommended from 4 weeks of age in Norway because of its low content in breast milk. In our study, the use of vitamin D supplements was higher in the intervention group compared with the control group. The recommendation of supplements [20] was discussed in the cooking classes. Even though the intervention group used vitamin D supplements to higher extent than the control group, there was no difference in vitamin D status between the groups. Such a relation has been reported by Gallo et al. showing effect of oral vitamin D supplementation on vitamin D status in infants [32]. We have not addressed possible confounders to this relation, such as sun exposure or ethnicity, which are both important for vitamin D status [33] and could be parts of explaining why we did not see any differences in vitamin D status.

Development of atherosclerosis and cardiovascular diseases starts at an early age and diet is one of the modifiable risk factors [10], indicating the importance of evaluating this intervention’s effect on serum lipids (risk factors for cardiovascular disease). The intervention group had higher HDL levels at 2 years of age than the control group. There were no differences in LDL/VLDL cholesterol between the two groups. A review by Cai et al. [34] concluded that childhood obesity prevention programmes had a desirable effect on LDL cholesterol and HDL cholesterol. However, just two of the included studies in their review used diet-alone intervention, and these showed no significant effect on HDL cholesterol; however, the combined interventions did [34]. The STRIP study is one of the more comparable studies to ours, as the authors started to intervene on diet at age 7 months, and reported improved lipid profiles (LDL cholesterol) after the intervention and even at the 20-year-follow up, especially in boys [35]. Our study is the first to show that diet-alone prevention early in life may improve HDL cholesterol and the first to quantify the effect of a cooking intervention in small children. The main differences in dietary intake between the intervention and control group were reduced intake of industrially produced porridge and dinners and the increased intake of home-made porridge. In Norway the most commonly used home-made porridge is oatmeal porridge [36]. Oats have previously been found to have cholesterol-lowering properties [37]. Oats are rich in dietary fibre, especially beta-glucan, which is suggested to be primarily responsible for their cholesterol-improving property [37].

## Strengths and limitations

The study has several strengths, the main one being that it is a randomized controlled trial, with the potential to establish cause and effect. Furthermore, the effect of the intervention was observed by objective measures of lipid status.

There are limitations to the present study. Power calculations estimated that we needed approximately 80 parent/child pairs in each group to find differences in food intake. Recruitment of participants was difficult and we spent more than 2 years recruiting 143 participants. However, there was a high attrition rate, resulting in our final participant numbers being 39 and 20 pairs in the intervention and control groups, respectively. One could also argue that the time of intervention is a bit late to address the introduction of solid foods, as most infants are introduced to solids before the age of 6 months. However, because the intervention included that the infants should taste the prepared dishes, we did not want to disrupt the possibility of exclusively breastfeeding the children for 6 months, which was the recommendation in Norway at the time [20]. Furthermore, there were limitations with the dietary assessment methods. First, the questionnaires were not validated, although similar questionnaires are used in the national dietary survey in Norway [38]. Second, the dietary habits were self-reported. Self-reporting diet may lead to an overreporting of healthy food items or habits and under-reporting of unhealthy habits. Because we have no data on energy intake, we cannot estimate whether the participants have underestimated or overestimated the energy intake, which could have given an indication of misreporting. The results are still encouraging with regard to the intervention’s effect on lipid profile, and should be examined further in a larger randomized controlled trial.

## Conclusion

This study shows that a cooking intervention for parents when infants are being introduced to solids positively affects the child’s later diet and lipid profile, and possibly vitamin D status. Cooking classes for parents could be a part of the compulsory health care provision given to pregnant women and parents. However, as this study was limited by its low participation, it should be replicated in a larger study.
